# Inflammatory Biomarkers Decay After First‐Line Antiretroviral Therapy Initiation With Dolutegravir/Lamivudine or Bictegravir/Emtricitabine/Tenofovir Alafenamide in Persons With HIV: A Substudy of a Randomized Clinical Trial

**DOI:** 10.1002/hsr2.71584

**Published:** 2026-03-04

**Authors:** Analuz Fernandez, Sofía Scevola, Raul Rigo Bonin, Maria Saumoy, Arkaitz Imaz, Daniel Podzamczer, Juan Tiraboschi

**Affiliations:** ^1^ Bellvitge University Hospital, L'Hospitalet de Llobregat Barcelona Spain; ^2^ Pharmacology Service, Bellvitge University Hospital, L´Hospitalet de Llobregat Barcelona Spain

**Keywords:** antiretroviral therapy, bictegravir, dolutegravir, HIV, inflammatory biomarkers

## 1 Background

Antiretroviral therapy (ART) has transformed human immunodeficiency virus (HIV) infection into a chronic condition. Triple‐drug therapy has long been the standard of care; however, dual therapy with dolutegravir/lamivudine (DTG/3TC) has demonstrated noninferiority in randomized clinical trials, both in treatment‐naive and experienced individuals [1, 2, 3]. Nonetheless, questions remain regarding the effect of DTG/3TC on underlying immuno‐virological dynamics beyond plasma viral suppression.

Persistence of HIV in reservoirs such as central nervous system (CNS), genital tract, lymph nodes, or gut mucosa‐associated lymphoid tissue [4, 5] is one of the barriers to HIV eradication and is associated with immune activation and chronic inflammation in people with HIV (PWH) [6]. Inadequate ART penetration in these compartments may lead to local viral replication, occasionally inducing a low‐level “viral escape” [7]. This may partly explain persistent immune activation despite viral suppression in plasma [8].

Immune activation and chronic inflammation in PWH are associated with higher risk of AIDS‐ and non‐AIDS‐related events, and all‐cause mortality [9, 10, 11, 12]. This may also contribute to the HIV pathogenesis, the accentuated and accelerated ageing observed in PWH, and earlier onset of comorbidities such as cardiovascular disease, neurocognitive decline, and cancer [10, 11, 12, 13].

Elevated levels of inflammatory and coagulation markers such as high‐sensitivity C‐reactive protein (hs‐CRP), interleukin‐6 (IL‐6), soluble CD14 (sCD14), soluble CD163 (sCD163), and D‐dimer are linked to increased complications and mortality in PWH [9, 10, 11, 12]. While ART reduces several biomarkers levels, others like IL‐6, sCD14, sCD163, TNF‐α, sVCAM‐1, FABP‐2, and D‐dimer remain higher than the general population [12, 13, 14, 15]. Moreover, sVCAM‐1 and FABP‐2 have been associated with morbidity and mortality [15]. However, whether DTG/3TC or BIC/FTC/TAF leads to more rapid or greater reductions in these inflammatory markers in ART‐naïve PWH remains unclear.

This study aims to assess whether there are differences in the decline of inflammatory biomarkers with two current first‐line regimens: the dual DTG + 3TC and the triple Bictegravir/Emtricitabine/Tenofovir alafenamide (BIC/FTC/TAF). Understanding these differences may help to clarify immunovirological dynamics beyond viral suppression, which can impact the long‐term health of PWH.

## 2 Methods

We conducted a substudy within an open‐label, multicenter, randomized pilot clinical trial (The DOLLARS Study), which included ART‐naive males to evaluate HIV viral load decay over 24 weeks in blood plasma, seminal plasma, and rectal fluid [16]. Asymptomatic, ART‐naïve males with plasma HIV‐1 RNA >1000 copies/mL and < 500,000 copies/mL, CD4 cell count > 200/μL, negative for Hepatitis B virus (HBV), and with the absence of mutations associated with resistance to the study treatments were eligible. Individuals with opportunistic infections, sepsis, infections in general, or active cancer were excluded. Baseline characteristics of participants are summarized in Table 1.

**Table 1:** Baseline characteristics of participants.

Baseline characteristics	DTG + 3TC *n* = 16	BIC/F/TAF *n* = 8	*p*‐value
Age (years), median (range)	31 (22–60)	32 (20–49)	0.83
CD4 count, cells/μL, median (range)	392 (331–463)	436.5 (216–716)	0.46
Blood plasma HIV‐1 RNA, log_10_ Copies/mL, (Median [Q1; Q3])	4.56 [4.22; 4.86]	4.48 [4.15; 5.04]	0.88
COVID‐19 (*n*, %)	1 (6.2)	0	1.00
Recent vaccination (*n*, %)	2 (12.5)	0	0.51
Smoke (*n*, %)	3 (18.7)	4 (50)	0.16
Use of recreative drugs (*n*, %)	5 (31.2)	4 (50)	0.63

Participants were randomized 2:1 to initiate BIC/FTC/TAF 50/200/25 mg oral dose (OD) or DTG 50 mg plus 3TC 300 mg OD, respectively. Randomization was stratified by baseline HIV‐1 viral load and CD4 T cell count. Blood samples were collected in EDTA‐containing tubes, and plasma samples were separated previous centrifugation at 2600 g for 15 min at 4°C, transferred to cryogenic vials, and stored at −80°C. hs‐CRP and IL‐6 levels were measured using a Cobas 8000 platform (Roche Diagnostics, Risch‐Rotkreuz, Switzerland); D‐dimer analyses were performed in the ACL TOP 500 analyzer using the HemosIL Dimer‐D HS reagent (Instrumentation Laboratory, Lexington, MA, USA). The other inflammatory biomarkers were determined using specific ELISA kits: sCD163 (Cat n.: MBS8807861; MyBioSource Inc., San Diego, CA), sCD14 (Cat n.: ELK8435; ELK Biotechnology CO. Ltd, Denver, CO, USA), and TNF‐α (Cat n.: EK0525), VCAM‐1 (Cat n.: EK0537), and FABP‐2 (Cat n.: EK1410) from Boster Bio, Pleasanton, CA, USA. All these biomarkers, as well as the HIV‐1 RNA (LOQ 20 copies/mL), were measured in plasma at baseline (BL), Days 3, 7, 14, and 28, and Weeks 12 and 24.

The linear relationship between the different inflammatory markers was studied through a correlation matrix. The mean of each inflammatory marker in each measurement (six visits) was reported in tables according to the treatment, as well as a graph to show the mean evolution of each marker in each visit according to the treatment. For modeling, mixed models with patient cluster effect were used for each marker, the dependent variable being the values of said marker and the independent variables being time and treatment. The interaction between the independent variables was also studied. The difference in evolution compared to baseline at each time point was compared by calculating the effect size using Cohen's D. Whenever possible, point estimates were accompanied by a 95% confidence interval. Statistical analyzes were performed with the R program in version 4.2.1.

This study was conducted following the principles of good clinical practice, the provisions of the Declaration of Helsinki, and the requirements of the Spanish regulatory authorities. The study protocol was approved by the NRTItutional Review Board at Bellvitge University Hospital. Written informed consent was provided by all participants before any study procedures were performed. As part of this substudy within a larger trial, the original DOLLARS consent form included permission for future research using stored samples and data. This study was registered at the EU Clinical Trials Registry (DOLLARS Study, EudraCT 2019‐004109‐28).

## 3 Results

Twenty‐five participants were included: 16 in the DTG/3TC arm and 9 in the BIC/FTC/TAF arm. We included an additional subject that was originally excluded from the main study analysis, as he met acute HIV infection criteria.

Median (range) age was 32 (20–60) years; BL CD4 count and HIV‐1 RNA in plasma were 398 (216–716) cells/μL and 4.56 (3.09–5.65) log10 copies/mL, respectively. No statistically significant differences were observed between treatment groups in HIV‐1 RNA at BL. By Week 24, viral load < 20 copies/mL was achieved in 78% (7/9) in the triple therapy group and 88% (14/16) in the dual therapy group, with no statistically significant difference between groups (*p* = 0.6016).

Prospective biomarker plasma values are detailed in Table 2. hs‐CRP difference from baseline to Week 24 was −0.14 mg/L in the DTG/3TC group and −3.92 mg/L for the BIC/FTC/TAF group (*p*: 0.37). Due to great variability in IL‐6 values, we analyzed the number of subjects presenting with results above the median value of 3.5 ng/L from baseline to Week 24. At the end of the study, one subject in each group remained above this value (3.7 ng/L in the DTG/3TC group and 3.8 ng/L in the BIC/FTC/TAF group) (*p*: 1.00). D‐dimer change from baseline to Week 24 was −30.7 and −72.2 µg/L in the DTG/3TC and BIC/FTC/TAF groups, respectively (*p* = 0.47). sCD163 change from baseline to week was −220.1 and −233.7 ng/L in the DTG/3TC and BIC/FTC/TAF groups respectively (*p* = 0.87). sCD14 decline was −134.5 and −242.8 µg/L in the DTG/3TC and BIC/FTC/TAF groups respectively (*p* = 0.64). Cell activation biomarkers VCAM‐1 and FABP‐2 changes from baseline to Week 24 were −4.5 and −296.1 ng/L in the DTG/3TC group and −47.4 and −366.7 ng/L in the BIC/FTC/TAF group (*p* = 0.30 and 0.70). TNF‐α reduction from baseline to Week 24 was −2.6 and −3.7 ng/L respectively (*p* = 0.68).

**Table 2:** Prospective plasma biomarker measurements in people with HIV receiving DTG+3TC or BIC/FTC/TAF.

Biomarker	Treatment group	Baseline	Day 3	Day 7	Day 14	Day 28	Week 12	Week 24
CRP (mg/L)	DTG + 3TC	1.7 [1; 2.5]	2.3 [1; 3.7]	3.8 [0.5; 7.1]	2.6 [0.7; 4.4]	1.9 [0.8; 3.0]	2 [0.9; 3.0]	1.6 [1.03; 2.21]
	BIC/FTC/TAF	7.8 [0; 17.4]	4.6 [0; 9.7]	2.9 [0.7; 5.]	1.6 [0.7; 2.4]	1.1 [0.4; 1.8]	1.2 [0.2; 2.2]	1.43 [0.15; 2.72]
*p*‐value			0.1	0.08	0.11	0.13	0.32	0.37
IL‐6 (subjects > 3.5 ng/L [%])	DTG + 3TC	1 (6.25%)	2 (12.5%)	3 (18.75%)	2 (14.29%)	3 (18.75%)	0 (0%)	1 (6.25%)
	BIC/FTC/TAF	2 (22.22%)	2 (22.22%)	2 (25%)	2 (22.22%)	1 (11.11%)	0 (0%)	1 (14.2%)
DD (µg/L)	DTG + 3TC	79.6 [49.1; 110.2]	119 [74.1; 163.9]	99 [60.5; 137.4]	82.2 [42.8; 121.7]	59.6 [36.9; 82.3]	51.9 [34.9; 68.9]	48.9 [33.9; 63.9]
	BIC/FTC/TAF	186.2 [53.3; 319]	214.6 [54.2; 375]	183 [50.5; 315.4]	142.2 [42.9; 241.5]	141.3 [41.9; 240.7]	121.5 [36.1; 206.8]	111.1 [15.5; 206.7]
*p*‐value			0.6	0.29	0.23	0.41	0.75	0.47
sCD14 (µg/L)	DTG + 3TC	2231.5 [1810.5; 2652.5]	2339.3 [1913.35; 2765.2]	2240.7 [1858; 2623.4]	2262.9 [1822; 2703.7]	2261.2 [1953.9; 2568.6]	2271.2 [1931.3; 2611]	2097.02 [1825.2; 2368.7]
	BIC/FTC/TAF	2518.1 [1874.3; 3162]	2581.9 [2015.2; 3148.5]	2584.6 [2193.9; 2975.2]	2294.9 [1777.1; 2812.7]	2390.1 [1826.2; 2954]	2343.5 [1794.5; 2892.6]	2360.8 [1996.5; 2725]
*p*‐value			0.65	0.57	0.08	0.32	0.710	0.64
sCD163 (ng/L)	DTG + 3TC	926.8 [773.3; 1080.3]	946 [819.8; 1072.2]	918.3 [836; 1000.6]	860.1 [776.26; 944]	808 [706.3; 909.7]	740.61 [628.92; 852.3]	706.7 [606.02; 807.3]
	BIC/FTC/TAF	1010.5 [705.8; 1315.3]	985.2 [723.7; 1246.8]	973.1 [745.8; 1200.4]	886.3 [732.4; 1040.2]	812.44 [653.4; 971.4]	735.6 [506.7; 964.5]	759.1 [451.8; 1066.5]
*p*‐value			0.34	0.38	0.41	0.33	0.7	0.83
TNF‐α (ng/L)	DTG + 3TC	20.8 [16.2; 25.5]	21.7 [16.7; 26.7]	20.2 [15.9; 24.6]	20.6 [16.1; 25]	21.2 [16.9; 25.6]	19.6 [15; 24.2]	18.2 [14.6; 21.8]
	BIC/FTC/TAF	22.8 [16.1; 29.5]	22.3 [15.6; 29]	23.1 [17.1; 29.1]	20.7 [15.8; 25.6]	21 [16.2; 25.8]	21.1 [17.5; 24.7]	19.3 [15.5; 23.1]
*p*‐value			0.07	0.73	0.18	0.16	0.8	0.68
FABP‐2 (ng/L)	DTG + 3TC	2078.56 [1787.09; 2370.03]	2161.56 [1857.12; 2466.01]	2121.12 [1788.8; 2453.45]	1998.5 [1679.16; 2317.84]	1977.06 [1680.58; 2273.55]	1861.13 [1535.06; 2187.2]	1782.3 [1474.8; 2089.9]
	BIC/FTC/TAF	2511.22 [1696.41; 3326.04]	2466.78 [1751.74; 3181.82]	2488.12 [1693.52; 3282.73]	2331.11 [1735.74; 2926.48]	2495.67 [1913.22; 3078.11]	2268.38 [1606.6; 2930.15]	2072.4 [1416.5; 2728.3]
*p*‐value			0.21	0.05	0.34	0.57	0.43	0.79
VCAM‐1 (µg/L)	DTG + 3TC	452 [376.1; 527.9]	447.7 [375.7; 519.8]	422 [356.1; 487.9]	437.4 [354.7; 520.1]	428.3 [353.8; 502.8]	453.6 [364.2; 542.9]	447.5 [366.6; 528.5]
	BIC/FTC/TAF	507.1 [378.5; 635.7]	499.8 [387.3; 612.4]	473.1 [353.6; 592.6]	468.8 [366.9; 570.8]	462.1 [368; 556.2]	427.2 [325.6; 528.8]	431.4 [326.3; 536.5]
*p*‐value			0.91	0.98	0.81	0.61	0.2	0.37

Two subjects in the BIC/FTC/TAF had no available inflammatory biomarkers sample at Week 24. By that timepoint, one of them was virologically suppressed and the other one had plasma HIV‐1 RNA of 37 copies/mL.

We found no statistically significant interaction between biomarker results and some confounding factors, such as baseline HIV‐1 RNA > 100,000 copies/mL, smoking, recent vaccination at screening, reported adverse events during follow‐up and COVID‐19 infection during the study.

All biomarker values and statistical comparisons are detailed in the Supporting Information: Supplementary Appendix.

## 4 Discussion

In this substudy, randomized ART with either DTG/3TC or BIC/FTC/TAF resulted in a decrease of Inflammatory biomarkers from baseline, throughout study time points, and up to Week 24, with no statistically significant differences between these dual or triple strategies.

Virological suppression was achieved in most participants in both arms, with no statistically significant difference observed between groups.

Data from the pivotal GEMINI‐1 and GEMINI‐2 studies confirmed the efficacy and safety of DTG/3TC compared to standard 3‐drug regimens in ART‐naïve individuals [1]. However, as previously noted, concerns remain about whether using two versus three drugs might reduce antiviral activity in certain tissues. This could contribute to persistent inflammation, potentially leading to immune exhaustion and both AIDS and non‐AIDS‐related complications. In this context, investigators of GEMINI‐1 and GEMINI‐2 reported minimal or no changes in key inflammatory markers (IL‐6 and hs‐CRP) between the two treatment groups from baseline to Week 144 [17].

Furthermore, an observational, prospective study evaluated changes in hs‐CRP, IL‐6, IL‐8, and TNF‐α in serum samples from 71 ART‐naïve subjects initiating DTG/3TC or DTG/3TC plus Abacavir (ABC). The median decrease of all inflammatory markers reported after 6 months was comparable between groups [18]. Investigators from the same group evaluated inflammatory biomarkers in serum from a nonrandomized cohort of PWH initiating either dual‐combination (DTG/3TC) or BIC/FTC/TAF. Again, a similar reduction in serum concentration of hsCRP, IL‐6, IL‐8, and TNF‐α was observed in both treatment groups [19].

More recently, an open‐label, randomized clinical trial was published. Similar to our study, participants were assigned to initiate their antiretroviral treatment with either a dual (DTG/3TC) or a triple (DTG + 3TC/TAF) ART. Biomarkers from peripheral blood (IL‐6, IL‐10, D‐dimer, sCD14, hs‐CRP, FABP2), as well as cell activation (HLA‐DR/CD38) and exhaustion (PD‐1/TIGIT), markers were evaluated throughout 48 weeks. Investigators reported a similar reduction in evaluated parameters of HIV persistence as well as in other immune‐associated markers [20].

Our research directly compares head‐to‐head two currently recommended first‐line regimens in ART‐naïve individuals. Alongside with the findings from the main DOLLARS study, it underscores the importance of achieving rapid HIV suppression in the so‐called “viral sanctuaries” beyond the peripheral blood compartment [16] as viral suppression is the primary driver of inflammation reduction, regardless of the number of antiretroviral drugs used. A recent study conducted in ART‐naïve individuals also showed similar reductions in HIV reservoir size between two‐drug and three‐drug regimens [20], supporting that second‐generation INSTI‐based regimens combined with one or two NRTIs appear to be potent enough to achieve this goal.

Some limitations need to be pointed out, and it is necessary to interpret these results with caution. First, the limited sample size and the short duration of the follow‐up. Second, a high intrasubject variability (intraclass coefficient > 0.7) was found in all the biomarkers analyzed. Third, even though we selected a pool of the most studied serum inflammation surrogate biomarkers, their clinical meaning is still no universal accepted. Finally, all our participants were male.

In conclusion, our results suggest that initiating antiretroviral treatment with DTG/3TC or BIC/FTC/TAF resulted in rapid viral suppression and reduced inflammation with no differences between strategies. Future research should aim to confirm these results in larger cohorts with longer follow‐up, and investigate the clinical implications of persistent immune activation despite virological suppression in blood with current recommended ART.

## Author Contributions

**Analu Fernandez:** data curation, investigation, validation, visualization, writing – original draft, writing – review and editing. **Sofía Scevola:** methodology, writing – original draft. **Raul Rigo Bonin:** investigation. **Maria Saumoy:** supervision, validation, visualization. **Arkaitz Imaz:** resources, supervision, validation, visualization. **Daniel Podzamczer:** methodology, resources, supervision, validation, visualization. **Juan Tiraboschi:** formal analysis, investigation, methodology, project administration, supervision, writing – review and editing.

## Conflicts of Interest

Sofía Scevola, Maria Saumoy, Daniel Podzamczer, and Juan Tiraboschi have received financial compensation for educational activities, as well as training courses, funds for research, travel grants, and nonfinancial support from Gilead Sciences, Janssen‐Cilag, Merck Sharp & Dohme, and ViiV Healthcare. Arkaitz Imaz has received financial compensation for lectures, consultancy work, and educational activities, as well as funding for research, travel grants, and nonfinancial support from Gilead Sciences, Janssen‐Cilag, Merck Sharp & Dohme, Thera Technologies, and ViiV Healthcare. The remaining authors declare no conflicts of interest.

## Transparency Statement

The lead author, Juan Tiraboschi, affirms that this manuscript is an honest, accurate, and transparent account of the study being reported; that no important aspects of the study have been omitted; and that any discrepancies from the study as planned (and, if relevant, registered) have been explained.

## Supporting information

Supplementary appendix.

## Data Availability

The data that support the findings of this study are available in the Supporting Information S1 of this article.
