# Supplementary material for: Inflammatory Biomarkers Decay After First‐Line Antiretroviral Therapy Initiation With Dolutegravir/Lamivudine or Bictegravir/Emtricitabine/Tenofovir Alafenamide in Persons With HIV: A Substudy of a Randomized Clinical Trial
Source: Health Sci Rep. 2026 Mar 4;9(3):e71584. doi: 10.1002/hsr2.71584 (PMC12959470; doi:10.1002/hsr2.71584)

Supplementary appendix.

BIC/FTC/TAF or Biktarvy: Bictegravir/emtricitabine/tenofovir alafenamide.

DTG/3TC: Dolutegravir/Lamivudine.

1. **Flowchart.**


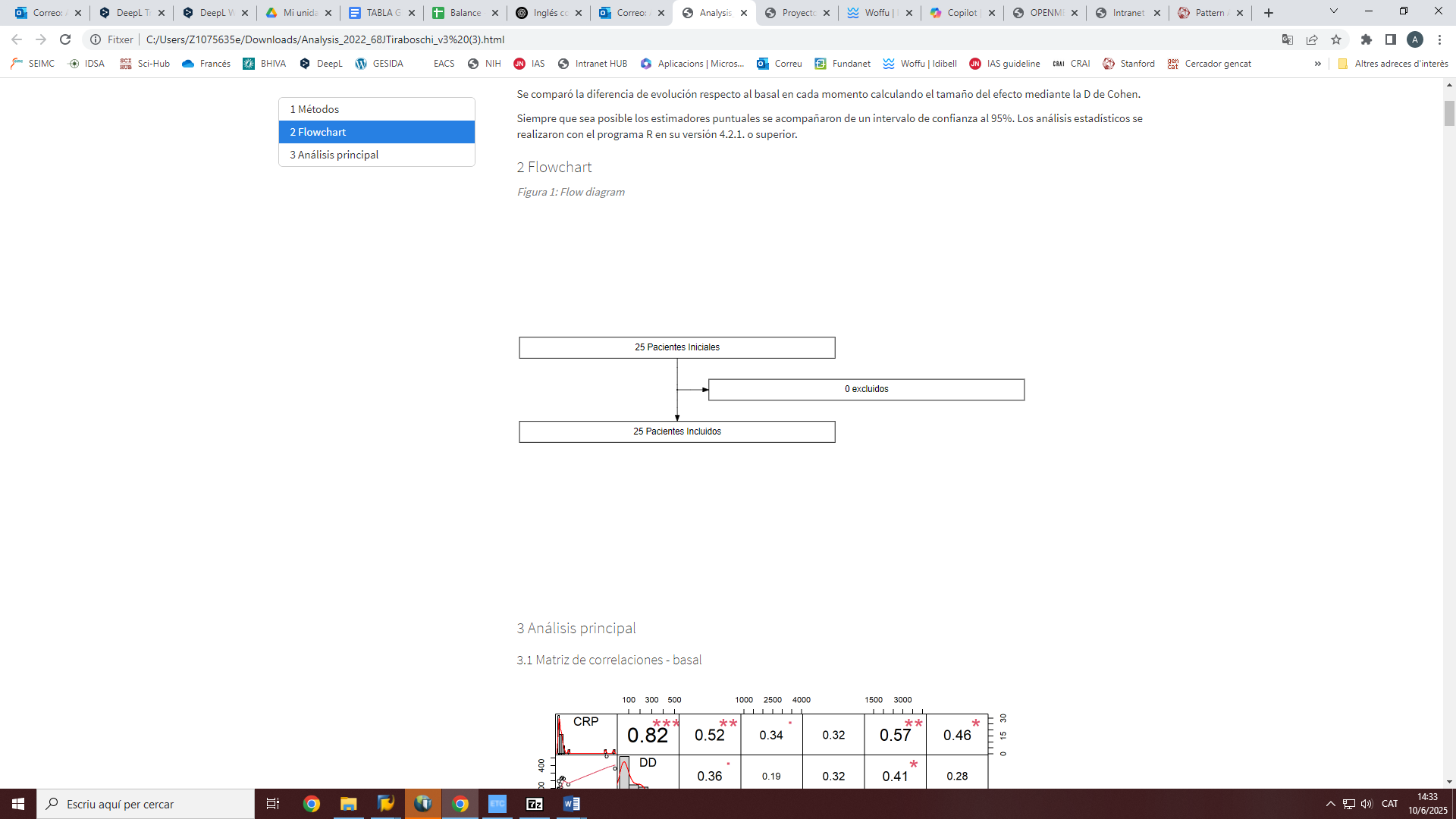


25 individuals were included.

0 excluded.

25 Initial candidates.

1. **C-reactive protein (CRP).**
   1. Table.

| **CRP, Media [IC95%]** | | | | | | | |
| --- | --- | --- | --- | --- | --- | --- | --- |
| **Treatment** | **Baseline** | **Day 3** | **Day 7** | **Day 14** | **Day28** | **Week 12** | **Week 24** |
| DTG+3TC | 1.76 [1.01; 2.52] | 2.38 [1.01; 3.74] | 3.87 [0.56; 7.17] | 2.61 [0.79; 4.43] | 1.94 [0.86; 3.03] | 1.93 [0.93; 2.93] | 1.62 [1.03; 2.21] |
| Biktarvy | 7.8 [0; 17.49] | 4.67 [0; 9.75] | 2.9 [0.77; 5.02] | 1.61 [0.76; 2.47] | 1.1 [0.4; 1.8] | 1.24 [0.28; 2.2] | 1.43 [0.15; 2.72] |

- 1. Differences regard to the baseline.

|  | **DTG/3TC** | **BIC/FTC/TAF** | **p.**  **overall** |
| --- | --- | --- | --- |
|  | *N=16* | *N=9* |  |
| Day 3 - Baseline, Mean (standard deviation) | 0.61 (1.46) | -3.13 (6.09) | 0.105 |
| Day 7 - Baseline, Mean (standard deviation) | 2.10 (5.08) | -5.81 (11.0) | 0.086 |
| Day 14 - Baseline, Mean (standard deviation) | 0.83 (2.16) | -6.19 (11.8) | 0.114 |
| Day 28 - Baseline, Mean (standard deviation) | 0.18 (2.11) | -6.70 (12.5) | 0.138 |
| Week 12 - Baseline, Mean (standard deviation) | 0.16 (1.91) | -3.51 (9.67) | 0.322 |
| Week 24 - Baseline, Mean (standard deviation) | -0.14 (1.55) | -3.92 (10.3) | 0.373 |

- 1. Evolution graphic.


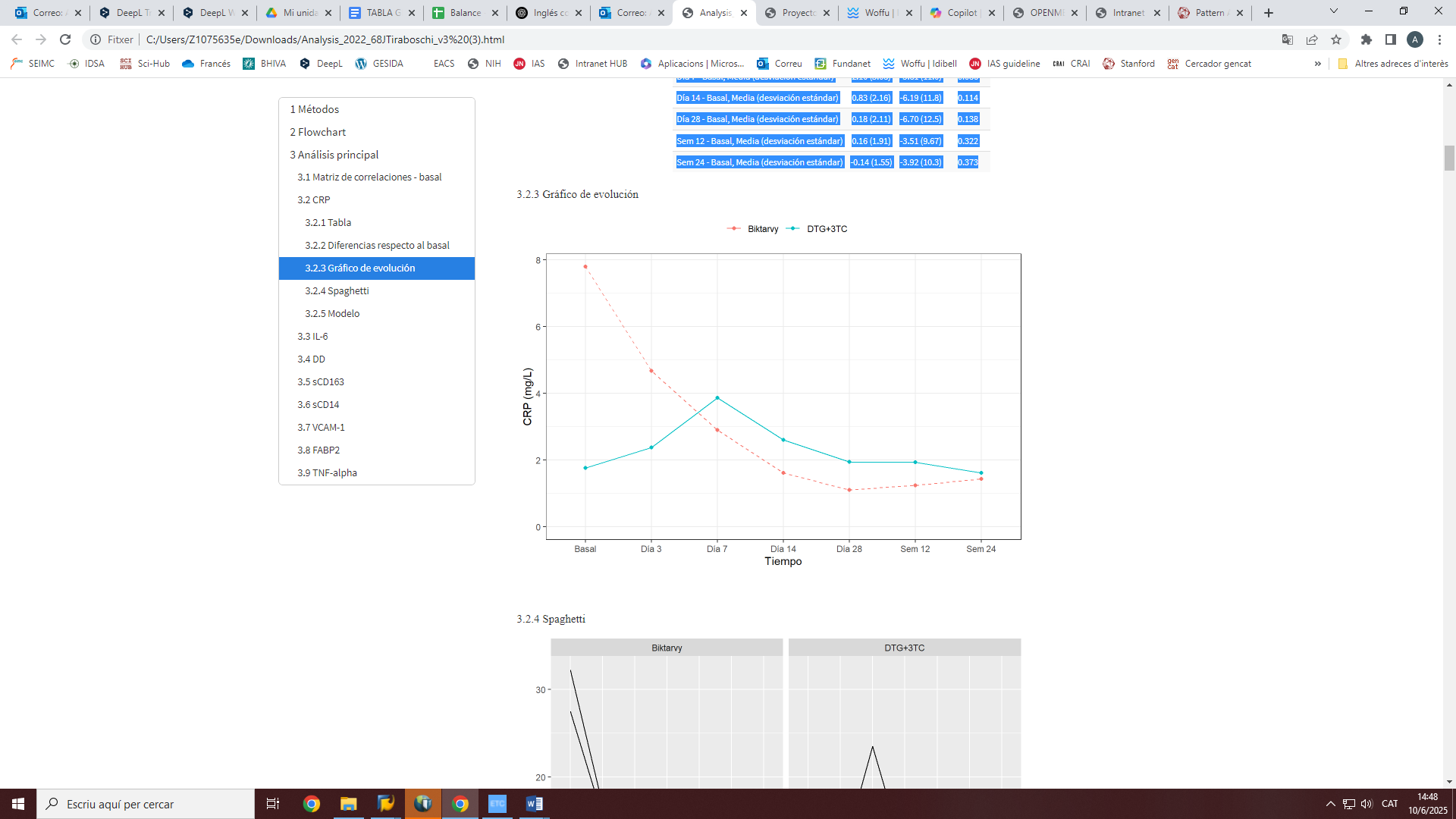


1. **Interleuquine-6 (L6)**
   1. Differences regard to the baseline.

|  | **Baseline** | **Day 3** | **Day 7** | **Day 14** | **Day 28** | **Week 12** | **Week 24** | **N** |
| --- | --- | --- | --- | --- | --- | --- | --- | --- |
| DTG+3TC, N (%) | 1 (6.25%) | 2 (12.5%) | 3 (18.75%) | 2 (14.29%) | 3 (18.75%) | 0 (0%) | 1 (6.25%) | 16 |
| Biktarvy, N (%) | 2 (22.22%) | 2 (22.22%) | 2 (25%) | 2 (22.22%) | 1 (11.11%) | 0 (0%) | 1. (14.29%) | 9 |

- 1. Evolution graphic.


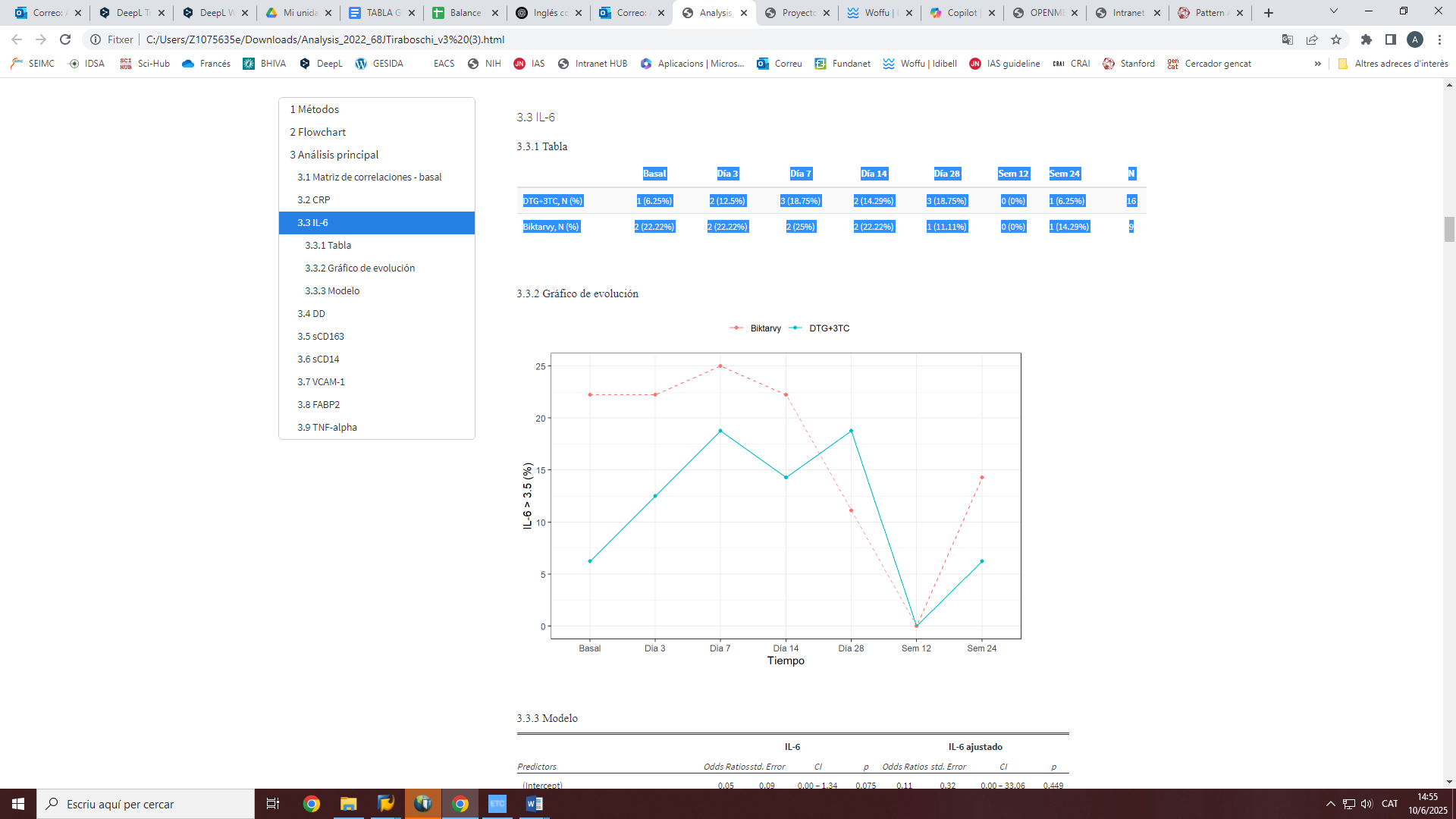


1. **D Dimer** **(DD).**
   1. Table.

| **DD, Mean [CI95%]** | | | | | | | | |
| --- | --- | --- | --- | --- | --- | --- | --- | --- |
| **Treatment** | **Baseline** | **Day 3** | **Day 7** | **Day 14** | **Day 28** | **Week 12** | **Week 24** |  |
| DTG+3TC | 79.69 [49.11; 110.26] | 119.06 [74.18; 163.95] | 99 [60.51; 137.49] | 82.29 [42.87; 121.7] | 59.62 [36.93; 82.32] | 51.56 [35.76; 67.37] | 48.94 [33.95; 63.93] |  |
| BIC/FTC/TAF | 186.22 [53.38; 319.06] | 214.67 [54.26; 375.07] | 183 [50.51; 315.49] | 142.22 [42.92; 241.52] | 141.33 [41.96; 240.7] | 121.5 [36.12; 206.88] | 111.14 [15.58; 206.7] |  |

- 1. Differences regard to the baseline.

|  | **DTG+3TC** | **BIC/FTC/TAF** | **p.overall** |
| --- | --- | --- | --- |
|  | *N=16* | *N=9* |  |
| Day 3 - Baseline, Mean (standard deviation) | 39.4 (33.8) | 28.4 (56.3) | 0.605 |
| Day 7 - Baseline, Mean (standard deviation) | 19.3 (54.0) | -22.38 (98.5) | 0.293 |
| Day 14 - Baseline, Mean (standard deviation) | -4.07 (45.6) | -44.00 (88.2) | 0.236 |
| Day 28 - Baseline, Mean (standard deviation) | -20.06 (31.4) | -44.89 (84.4) | 0.417 |
| Week 12 - Baseline, Mean (standard deviation) | -28.12 (40.6) | -43.12 (130) | 0.758 |
| Week 24 - Baseline, Mean (standard deviation) | -30.75 (47.0) | -72.29 (141) | 0.472 |

- 1. Evolution graphic.


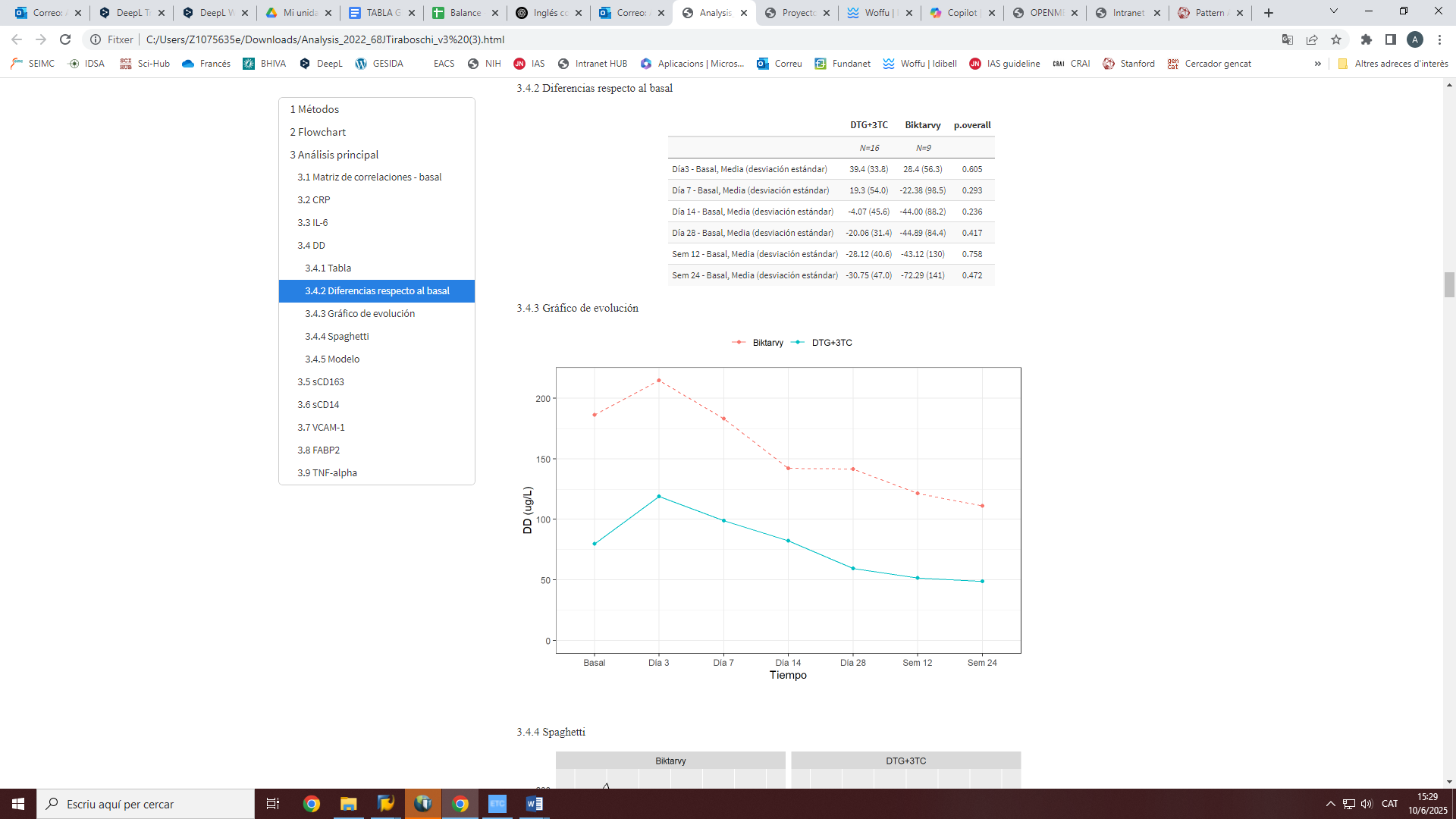


1. **Soluble CD163 (sCD163)**
   1. Table.

|  | **sCD163, Mean [CI95%]** | | | | | | |
| --- | --- | --- | --- | --- | --- | --- | --- |
| **Treatment** | **Baseline** | **Day 3** | **Day 7** | **Day 14** | **Day 28** | **Week 12** | **Week 24** |
| DTG+3TC | 926.88 [773.39; 1080.37] | 946.03 [819.8; 1072.26] | 918.34 [836.09; 1000.6] | 860.14 [776.26; 944.03] | 808.04 [706.31; 909.77] | 741.51 [637.67; 845.36] | 706.7 [606.02; 807.38] |
| BIC/FTC/TAF | 1010.57 [705.8; 1315.34] | 985.28 [723.75; 1246.8] | 973.12 [745.81; 1200.44] | 886.39 [732.49; 1040.29] | 812.44 [653.4; 971.49] | 735.61 [506.7; 964.51] | 759.18 [451.84; 1066.52] |

- 1. Differences regard to the baseline.

|  | **DTG+3TC** | **BiC/FTC/TAF** | **p.overall** |
| --- | --- | --- | --- |
|  | *N=16* | *N=9* |  |
| Day 3 - Baseline, Mean (standard deviation) | 19.1 (92.1) | -25.29 (116) | 0.342 |
| Day 7 - Baseline, Mean (standard deviation) | -8.54 (180) | -83.26 (199) | 0.387 |
| Day 14 - Baseline, Mean (standard deviation) | -42.90 (206) | -124.18 (242) | 0.419 |
| Day 28 - Baseline, Mean (standard deviation) | -118.84 (178) | -198.12 (196) | 0.331 |
| Week 12 - Baseline, Mean (standard deviation) | -185.37 (214) | -213.72 (142) | 0.703 |
| Week 24 - Baseline, Mean (standard deviation) | -220.18 (217) | -233.76 (172) | 0.875 |

- 1. Evolution graphic.


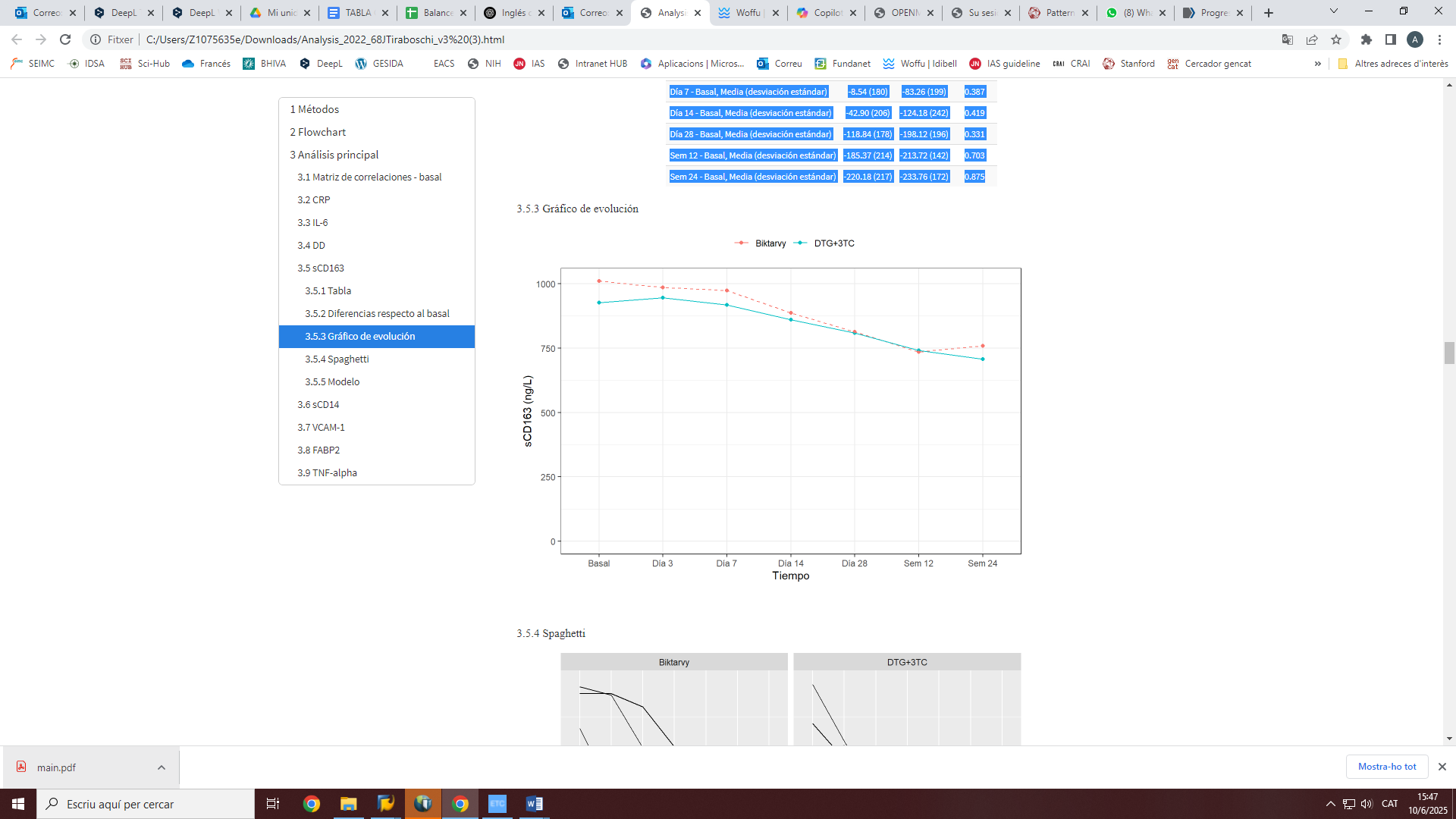


1. **Soluble CD14 (sCD14)**
   1. Table.

|  | **sCD14, Mean [CI95%]** | | | | | | |
| --- | --- | --- | --- | --- | --- | --- | --- |
| **Treatment** | **Baseline** | **Day 3** | **Day 7** | **Day 14** | **Day 28** | **Week12** | **Week 24** |
| DTG+3TC | 2231.56 [1810.56; 2652.56] | 2339.32 [1913.35; 2765.28] | 2240.72 [1858.02; 2623.41] | 2262.9 [1822.05; 2703.76] | 2261.26 [1953.91; 2568.61] | 2235.81 [1911.04; 2560.58] | 2097.02 [1825.29; 2368.76] |
| BIC/FTC/TAF | 2518.19 [1874.35; 3162.03] | 2581.93 [2015.29; 3148.57] | 2584.6 [2193.91; 2975.28] | 2294.91 [1777.13; 2812.7] | 2390.16 [1826.26; 2954.06] | 2343.58 [1794.52; 2892.65] | 2360.84 [1996.59; 2725.09] |

- 1. Differences regard to baseline.

|  | **DTG+3TC** | **BIC/FTC/TAF** | **p.overall** |
| --- | --- | --- | --- |
|  | *N=16* | *N=9* |  |
| Day 3 - Baseline, Mean (standard deviation) | 108 (263) | 63.7 (218) | 0.659 |
| Day 7 - Baseline, Mean (standard deviation) | 9.16 (445) | -127.74 (584) | 0.571 |
| Day 14 - Baseline, Mean (standard deviation) | 91.4 (398) | -223.28 (403) | 0.084 |
| Day 28 - Baseline, Mean (standard deviation) | 29.7 (398) | -128.03 (363) | 0.327 |
| Week 12 - Baseline, Mean (standard deviation) | 4.25 (440) | -55.26 (319) | 0.710 |
| Week 24 - Baseline, Mean (standard deviation) | -134.54 (572) | -242.83 (466) | 0.640 |

- 1. Evolution graphic.


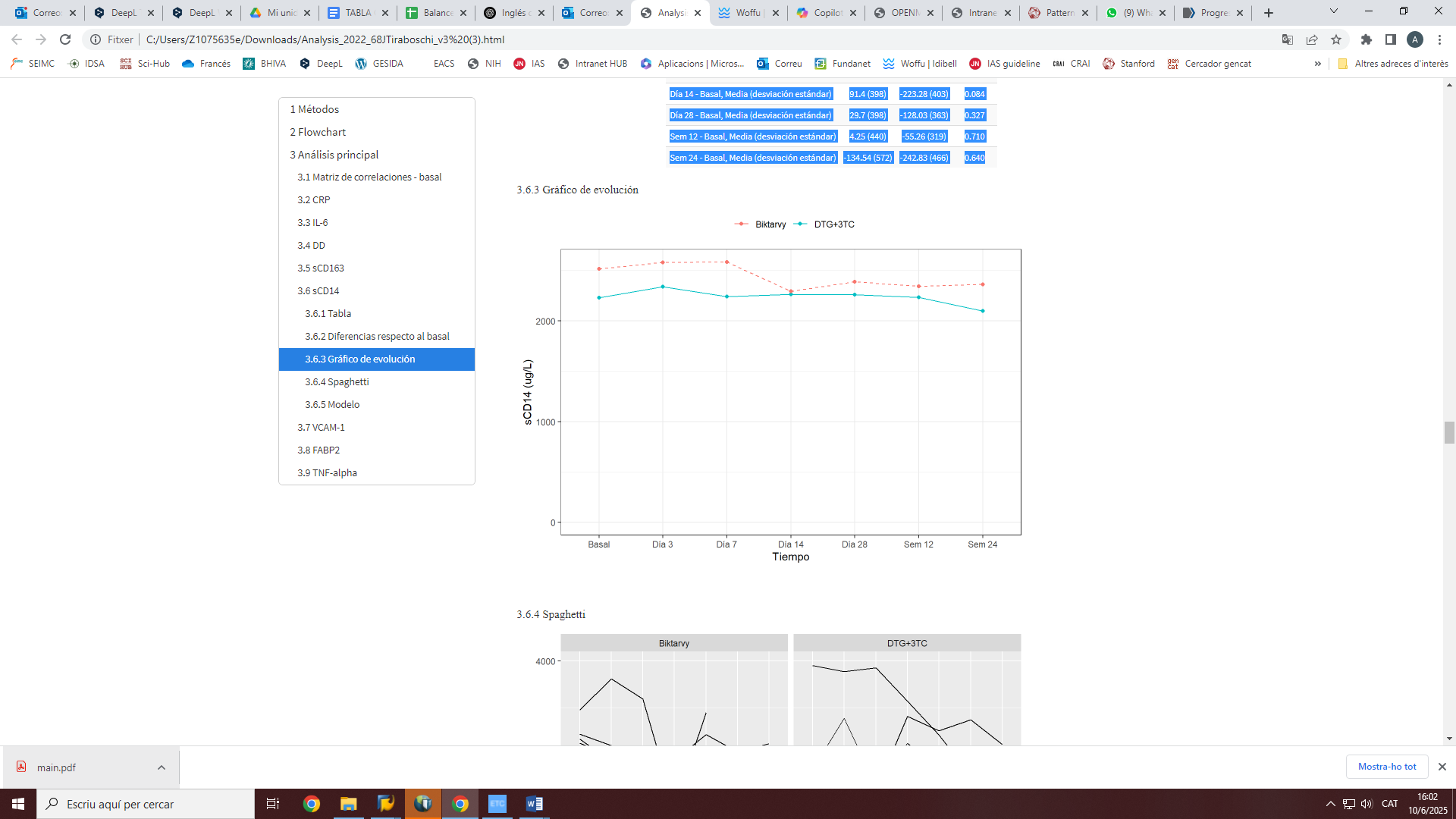


1. **VCAM-1**
   1. Table.

|  | **VCAM-1, Mean [CI95%]** | | | | | | |
| --- | --- | --- | --- | --- | --- | --- | --- |
| **Treatment** | Baseline | Day 3 | Day 7 | Day14 | Day 28 | Week 12 | Week 24 |
| **DTG/3TC** | 452.06 [376.19; 527.93] | 447.75 [375.7; 519.8] | 422.06 [356.16; 487.97] | 437.43 [354.7; 520.16] | 428.38 [353.87; 502.88] | 446.31 [361.79; 530.83] | 447.56 [366.61; 528.52] |
| **BIC/FTC/TAF** | 507.11 [378.5; 635.72] | 499.89 [387.36; 612.41] | 473.12 [353.65; 592.6] | 468.89 [366.91; 570.86] | 462.11 [368.02; 556.2] | 427.25 [325.62; 528.88] | 431.43 [326.31; 536.55] |

- 1. Differences regard to baseline.

|  | **DTG/3TC** | **BIC/FTC/TAF** | **p.overall** |
| --- | --- | --- | --- |
|  | *N=16* | *N=9* |  |
| Day 3 - Baseline, Mean (standard deviation) | -4.31 (36.9) | -7.22 (75.4) | 0.916 |
| Day 7 - Baseline, Mean (standard deviation) | -30.00 (84.0) | -29.12 (74.7) | 0.980 |
| Day 14 - Baseline, Mean (standard deviation) | -30.21 (56.2) | -38.22 (91.7) | 0.818 |
| Day 28 - Baseline, Mean (standard deviation) | -23.69 (67.6) | -45.00 (111) | 0.610 |
| Week 12 - Baseline, Mean (standard deviation) | -5.75 (70.6) | -60.00 (101) | 0.202 |
| Week 24 - Baseline, Mean (standard deviation) | -4.50 (74.8) | -47.43 (110) | 0.372 |

- 1. Evolution graphic.


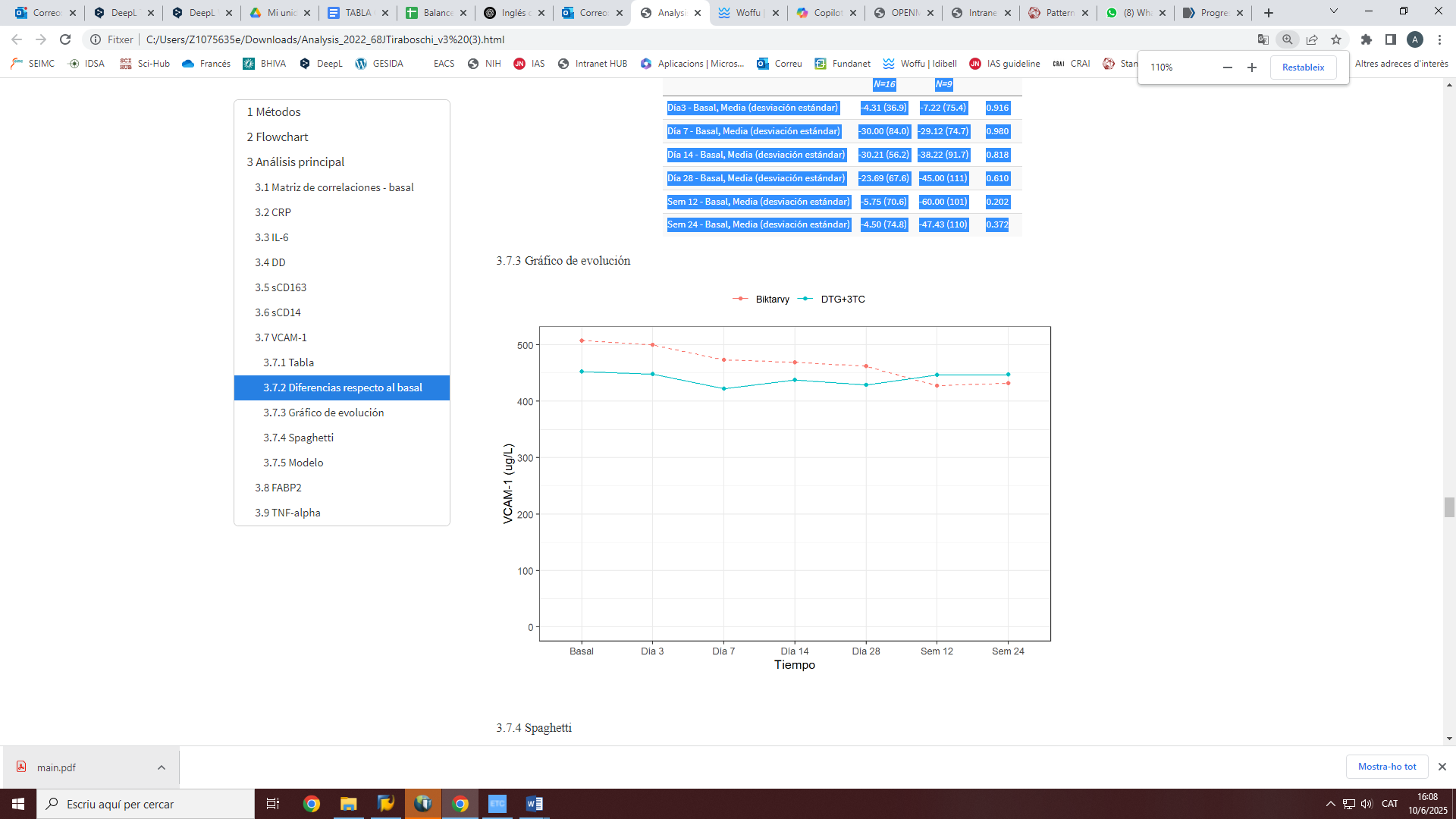


1. **FABP2.**
   1. Table.

|  | **FABP2, Mean [CI95%]** | | | | | | |
| --- | --- | --- | --- | --- | --- | --- | --- |
| **Treatment** | Baseline | Day 3 | Day 7 | Day14 | Day 28 | Week 12 | Week 24 |
| **DTG/3TC** | 2078.56 [1787.09; 2370.03] | 2161.56 [1857.12; 2466.01] | 2121.12 [1788.8; 2453.45] | 1998.5 [1679.16; 2317.84] | 1977.06 [1680.58; 2273.55] | 1897 [1584.39; 2209.61] | 1782.38 [1474.85; 2089.9] |
| **BIC/FTC/TAF** | 2511.22 [1696.41; 3326.04] | 2466.78 [1751.74; 3181.82] | 2488.12 [1693.52; 3282.73] | 2331.11 [1735.74; 2926.48] | 2495.67 [1913.22; 3078.11] | 2268.38 [1606.6; 2930.15] | 2072.43 [1416.52; 2728.34] |

- 1. Differences regard to the baseline.

|  | **DTG/3TC** | **BIC/FTC/TAF** | **p.overall** |
| --- | --- | --- | --- |
|  | *N=16* | *N=9* |  |
| Day 3 - Baseline, Mean (standard deviation) | 83.0 (183) | -44.44 (255) | 0.210 |
| Day 7 - Baseline, Mean (standard deviation) | 42.6 (215) | -156.50 (216) | 0.051 |
| Day 14 - Baseline, Mean (standard deviation) | -61.43 (155) | -180.11 (335) | 0.341 |
| Day 28 - Baseline, Mean (standard deviation) | -101.50 (213) | -15.56 (419) | 0.577 |
| Week 12 - Baseline, Mean (standard deviation) | -181.56 (198) | -46.38 (449) | 0.439 |
| Week 24 - Baseline, Mean (standard deviation) | -296.19 (272) | -366.71 (662) | 0.794 |

- 1. Evolution graphic.


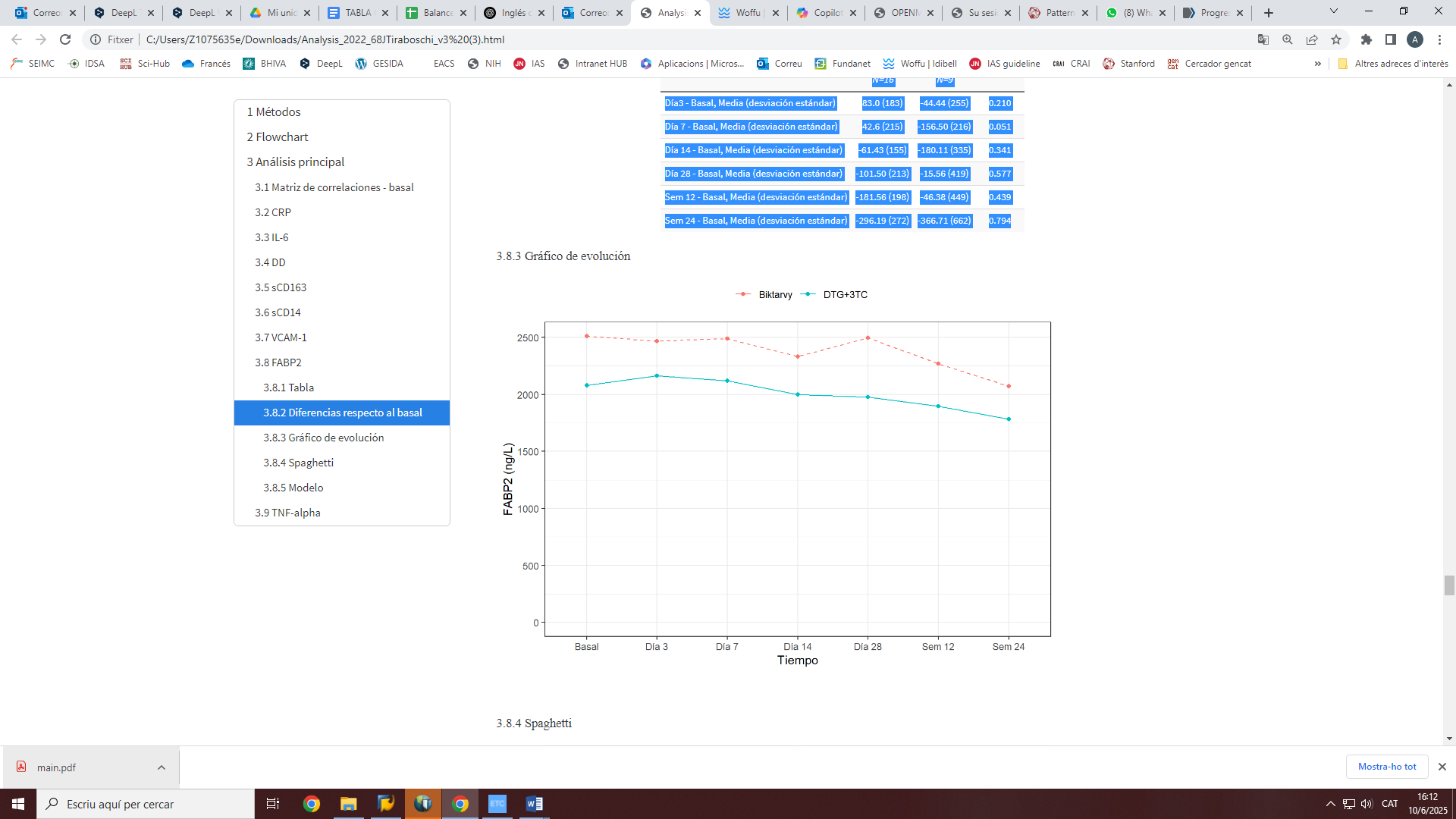


**7. alfa-Tumor Necrosis Factor (**TNF-α).

a. Table.

|  | **TNF-alpha, Mean [CI95%]** | | | | | | |
| --- | --- | --- | --- | --- | --- | --- | --- |
| **Tratamiento** | Baseline | Day 3 | Day 7 | Day14 | Day 28 | Week 12 | Week 24 |
| **DTG/3TC** | 20.89 [16.2; 25.57] | 21.73 [16.74; 26.73] | 20.28 [15.92; 24.64] | 20.61 [16.19; 25.03] | 21.27 [16.93; 25.6] | 20 [15.84; 24.17] | 18.27 [14.67; 21.88] |
| **BiC/FTC/TAF** | 22.86 [16.12; 29.59] | 22.33 [15.65; 29.01] | 23.15 [17.15; 29.15] | 20.74 [15.83; 25.66] | 21.06 [16.27; 25.85] | 20.26 [16.89; 23.63] | 19.34 [15.57; 23.12] |

b. Differences regard to the baseline.

|  | **DTG+3TC** | **BIC/FTC/TAF** | **p.overall** |
| --- | --- | --- | --- |
|  | *N=16* | *N=9* |  |
| Day 3 - Baseline, Mean (standard deviation) | 0.85 (2.39) | -0.52 (1.32) | 0.078 |
| Day 7 - Baseline, Mean (standard deviation) | -0.61 (2.77) | -1.05 (2.97) | 0.730 |
| Day 14 - Baseline, Mean (standard deviation) | 0.02 (3.93) | -2.11 (3.40) | 0.184 |
| Day 28 - Baseline, Mean (standard deviation) | 0.38 (4.16) | -1.80 (3.24) | 0.161 |
| Week 12 - Baseline, Mean (standard deviation) | -0.88 (5.44) | -1.41 (5.35) | 0.823 |
| Week 24 - Baseline, Mean (standard deviation) | -2.61 (4.99) | -3.70 (5.93) | 0.680 |

C. Evolution graphic.


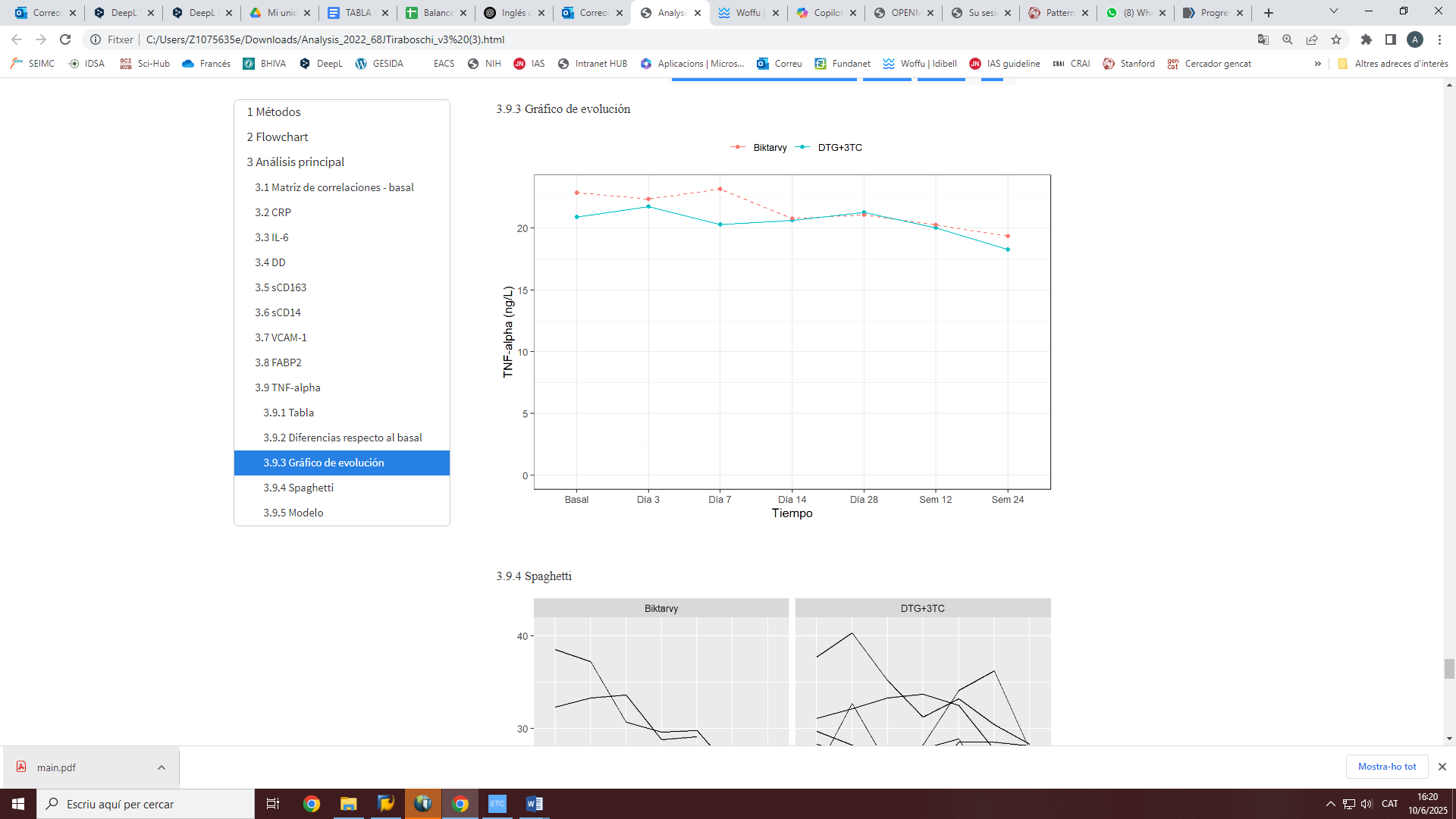

Supplement: Supplementary file 1 — Supplementary appendix. [file HSR2-9-e71584-s001.docx]
